# In vivo mouse cardiac hyperpolarized magnetic resonance spectroscopy

**DOI:** 10.1186/1532-429X-15-19

**Published:** 2013-02-18

**Authors:** Michael S Dodd, Vicky Ball, Rosalind Bray, Houman Ashrafian, Hugh Watkins, Kieran Clarke, Damian J Tyler

**Affiliations:** 1 Cardiac Metabolism Research Group, Department of Physiology, Anatomy & Genetics, University of Oxford, Oxford, UK; 2 Division of Cardiovascular Medicine, Radcliffe Department of Medicine, University of Oxford, Oxford, UK

## Abstract

**Background:**

Alterations in cardiac metabolism accompany many diseases of the heart. The advent of cardiac hyperpolarized magnetic resonance spectroscopy (MRS), via dynamic nuclear polarization (DNP), has enabled a greater understanding of the in vivo metabolic changes that occur as a consequence of myocardial infarction, hypertrophy and diabetes. However, all cardiac studies performed to date have focused on rats and larger animals, whereas more information could be gained through the study of transgenic mouse models of heart disease. Translation from the rat to the mouse is challenging, due in part to the reduced heart size (1/10^th^) and the increased heart rate (50%) in the mouse compared to the rat.

**Methods and Results:**

In this study, we have investigated the in vivo metabolism of [1-^13^C]pyruvate in the mouse heart. To demonstrate the sensitivity of the method to detect alterations in pyruvate dehydrogenase (PDH) flux, two well characterised methods of PDH modulation were performed; overnight fasting and infusion of sodium dichloroacetate (DCA). Fasting resulted in an 85% reduction in PDH flux, whilst DCA infusion increased PDH flux by 123%. A comparison of three commonly used control mouse strains was performed revealing significant metabolic differences between strains.

**Conclusions:**

We have successfully demonstrated a hyperpolarized DNP protocol to investigate in vivo alterations within the diseased mouse heart. This technique offers a significant advantage over existing in vitro techniques as it reduces animal numbers and decreases biological variability. Thus [1-^13^C]pyruvate can be used to provide an in vivo cardiac metabolic profile of transgenic mice.

## Background

Genetic mouse models have proved to be an invaluable tool in understanding and assessing the metabolic basis of heart disease. Transgenic and knockout (KO) mouse lines enable individual metabolic pathways to be probed, yielding valuable insights into the etiology of human heart disease. Mouse models such as the long chain acyl-CoA dehydrogenase-KO [1,2], peroxisome proliferator-activated receptor α-KO [3] and glucose transporter 1 [4] transgenic models, have increased our knowledge of the development and pathology of human heart diseases. The ability to target individual enzymes or complexes allows a greater understanding of their role and impact on the metabolic profile of heart disease and provide new angles for diagnosis and treatment.

Many techniques exist to assess the metabolic profile of a diseased or transgenic animal. Ultraviolet spectroscopy, used for over 70 years [5], allows the assessment of metabolite concentrations or enzyme activities in tissue homogenates. More recently, metabolomic techniques, including ^1^H-nuclear magnetic resonance (NMR), gas chromatography–mass spectrometry (MS) and liquid chromatography-MS have enabled detection of millimolar to sub-picomolar changes of metabolite levels/concentrations in disease [6,7]. However, these techniques all require the collection of tissue at specific time points, failing to allow serial measurements to be made in the same animal during the progression of a disease.

^13^C magnetic resonance spectroscopy (MRS) is particularly well suited to the study of metabolism in the heart due to the extensive range of metabolites that can be observed [8]. However, traditional ^13^C MRS suffers from an inherently low sensitivity, which necessitates long scan times and has limited in vivo cardiac ^13^C MRS studies [9,10]. These limitations have led to a reliance on ex vivo perfused heart models which prevents the study of the metabolic interplay between organs, such as the liver and heart.

A potential solution to the low sensitivity of ^13^C MRS has recently been developed with the advent of hyperpolarization, via dynamic nuclear polarization (DNP), whereby the sensitivity of ^13^C MRS can be increased more than 10,000-fold [11-13]. Hyperpolarized compounds can be infused in vivo and their metabolism visualized in real time [14]. To date, the most successful example of a DNP hyperpolarized molecule has been [1-^13^C]pyruvate [14-23] (recently reviewed in [24]). Pyruvate, the terminal molecule of glycolysis, can be processed by 3 main enzymes: pyruvate dehydrogenase (PDH), lactate dehydrogenase (LDH) and alanine aminotransferase (AAT). The control of these enzymes determines the relative contribution of glucose-derived carbon into the TCA cycle [25]. Relative fluxes through PDH into ^13^C]bicarbonate and ^13^CO_2_ have been shown to correlate with ex vivo PDH activity [19], whilst [1-^13^C]pyruvate’s processing into [1-^13^C]lactate and [1-^13^C]alanine provides a measure of the balance between glycolysis and glucose oxidation.

However, all cardiac studies performed so far have focused on rats or larger animals, whereas more information could be gained through the study of transgenic mouse models of heart disease. Translation from the rat to the mouse is challenging, due in part to the smaller heart size (~600 [26] vs. ~80 mg [27]), which will limit the measurable signal, and the higher heart rate (450 vs. 600 bpm [28]), which will increase motional artefacts and limit spectral resolution. In this study, we have developed an approach to investigate the metabolism of [1-^13^C]pyruvate in the in vivo mouse heart. To define the sensitivity of the developed method to detect alterations in PDH flux, mice were scanned in either the fed state, the fasted state (after removal of food for ~19 hours) or following an infusion of sodium dichloroacetate (DCA). Both fasting and DCA have been shown to modulate in vivo PDH flux in rat hearts [14,19]. Following evaluation of the developed technique, it was used to characterize the metabolic profile of a series of control mouse strains, C57BL/6, 129 SvEv and balb/c mice, as these are frequently used as breeding backgrounds for transgenic mouse lines [29,30].

The recent development of hyperpolarized ^13^C MRS has led to the first human trials of the technique in the study of prostate cancer at the University of California, San Francisco. This study aims to image changes in the production of [1-^13^C]lactate following injection of hyperpolarized [1-^13^C]pyruvate in the prostate, to detect alterations in tumour metabolism (Clinical trial identifier: NCT01229618 [31-33]). As recently reviewed by Schroeder et al. (2011) there are a wide variety of potential applications for hyperpolarized ^13^C MRS in the study of human cardiovascular metabolism [34]. As such, the aim of this work was to develop hyperpolarized MRS for mouse applications to provide a novel tool to assess the effect of genetic alterations in the in vivo heart and to aid in the translation of future findings to the clinic.

## Methods

[1-^13^C]Pyruvic acid and dichloroacetic acid were obtained from Sigma Aldrich (Sigma-Aldrich Company Ltd. Dorset, UK). Dichloroacetic acid was neutralized with sodium hydroxide, to form sodium DCA for injection into animals. Nineteen male C57BL/6 mice (~25 g, 15 week old), five male balb/c (~25 g, 15 week old), and four male Wistar rats (~250 g) were obtained from Harlan UK. Six male 129 SvEv (~25 g, 15 week old) were obtained from stable colonies at the University of Oxford. All animals were housed on a 12:12-h light–dark cycle and all fed animal studies were performed between 7 a.m. and 11 a.m., during the early absorptive (fed) state. Fasted animal scans were performed between 11 am and 1 pm following removal of food (minimum 19 hours). All investigations conformed to Home Office Guidance on the Operation of the Animals (Scientific Procedures) Act (HMSO) of 1986, to institutional guidelines and was approved by the University of Oxford Animal Ethics Review Committee.

### Animal handling

Anaesthesia was induced at 2.5-3% isoflurane in oxygen and nitrous oxide (4:1, total of 2 l/min). Anaesthesia was maintained by means of 2% isoflurane delivered to, and scavenged from, a nose cone during the experiment. A catheter (32 gauge needle) was introduced into the tail vein for intravenous infusion of hyperpolarized solutions and animals were then placed in a home-built animal-handling system, the same system was used for mice and rats [35]. ECG and respiration rate were monitored throughout the experiment and air heating was provided to maintain body temperature.

### Pyruvate polarization and dissolution

Approximately 40 mg of [1-^13^C]pyruvic acid doped with 15 mmol/L trityl radical (OXO63, Oxford Instruments, Abingdon, UK) and 3 μl Dotarem (1:50 dilution) (Guerbet, Birmingham, UK), was hyperpolarized in a polarizer (General Electric Prototype Polarizer, GE Healthcare, Amersham, UK), with 45 min of microwave irradiation, as previously described [11]. The sample was subsequently dissolved in a pressurized and heated alkaline solution, containing 60 mmol/L sodium hydroxide and 247 mmol/L EDTA dipotassium salt (Sigma-Aldrich), to yield a solution of 80 mM hyperpolarized sodium [1-^13^C]pyruvate with a polarization of ~30%, at physiological temperature and pH [14].

### Hyperpolarized ^13^C MRS protocol

**Figure 1:**
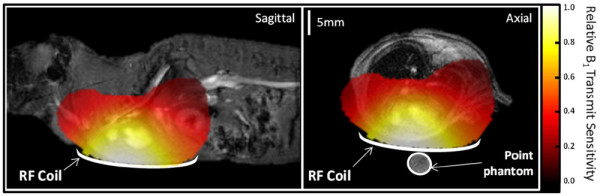
^**13**^**C field map of the mouse radiofrequency coil.** Correct positioning of the RF coil is vital for ensuring sensitivity to the back wall of the heart. This field map shows the sensitivity of the transmit/receive coil to ^13^C. The scale bar indicates relative B_1_ transmit profile, normalized to maximum of 1.

A circular ^13^C RF surface transmit/receive coil was built in-house and used for all mouse experiments in this study (radius 10 mm). To test the sensitivity profile of the coil, a field map was generated using an acetone phantom and then overlaid on top of an axial proton image of a mouse (Figure 1). The field map was calculated using a chemical shift imaging acquisition at multiple flip angle values (TR/TE, 1000/1.01 ms; flip angle, arrayed; Averages, 32; slice thickness, 20 mm; matrix, 8 × 8 × 512; field of view, 64 × 64 mm; zero-filled, 32 × 32 × 512). Sensitivity was detected at a distance sufficient to include signal from the back wall of the mouse heart (a depth of approximately 10 mm), confirming that the coil was appropriate for use in this study.

The ^13^C RF coil was placed over the mouse chest, localizing signal from the heart. Mice were positioned in a 7 T horizontal bore MR scanner interfaced to a direct-drive console (Varian Inc, Yarnton, UK). Using a 72 mm ^1^H volume transmit/receive RF coil (Rapid Biomedical, Rimpar Germany), a 3-plane FLASH localizer image was obtained to confirm the location of the heart at the magnet isocentre (TR/TE, 3.67/1.63 ms; flip angle, 24°; Averages, 16; slice thickness, 2 mm; matrix, 256 × 256; field of view, 51.2 × 51.2 mm). A fiducial marker containing water, positioned on top of the ^13^C RF coil, was used to ensure the correct position of the ^13^C RF coil over the heart. Using the ^1^H volume coil, an ECG-gated shim was used to reduce the proton linewidth to ~150 Hz.

Following dissolution, 0.15 ml of hyperpolarized pyruvate was injected over 10 s into the anesthetised mouse (dose of 0.48 mmol/kg), followed by a 0.05 ml flush of heparinized saline to clear the delivery line [21]. Sixty individual ECG-gated ^13^C MR pulse-acquire cardiac spectra were acquired over 1 min following injection (TR, 1 s; excitation flip angle, 15° at the front wall of the heart, assessed using the previously generated field map; sweep width, 8,012 Hz; acquired points, 2,048; frequency centred on the C1 pyruvate resonance).

### Development of mouse cardiac DNP using C57BL/6 mice

Male C57BL/6 mice (body weight (BW) = ~25 g) received [1-^13^C]pyruvate scans, in either the fed state (n = 7) or after fasting overnight for a minimum of 19 hours (n = 7). Another group of C57BL/6 mice (n = 5) received [1-^13^C]pyruvate scans after an infusion of DCA (30 mg/kg, in saline at pH 7.4). DCA (0.2 ml) was injected as a bolus into the tail vein cannula, followed by an infusion of 0.1 ml over 10 minutes. The end of the infusion was timed to allow immediate injection of hyperpolarized [1-^13^C]pyruvate. Due to the increased volume injected, mice were sacrificed immediately following the acquisition of the hyperpolarized spectroscopy data.

### Comparison with rats

Wistar rats have commonly been used as control animals for hyperpolarized cardiac studies. To perform a comparison between the fed C57BL/6 mice and fed Wistar rats (BW = ~250 g, dose of 0.32 mmol/kg), four male rats were also scanned using the methods set out in [16]. Briefly, rats were anesthetised using 2.5-3% isoflurane and maintained at 2%. A catheter was introduced into the tail vein for i.v. delivery of the hyperpolarized [1-^13^C]pyruvate solution. Following dissolution, 1 ml of hyperpolarized pyruvate was injected over 10 s into the anesthetised rat. Sixty individual ECG-gated ^13^C MR pulse-acquire cardiac spectra were acquired over 1 min after injection, using a custom built ^13^C loop butterfly RF coil (radius 20 mm) [16].

### Strain comparison

Male Balb/c (n = 5, BW = ~25 g) and male 129 SvEv (n = 6, BW = ~25 g) received [1-^13^C]pyruvate scans in the fed state to compare the metabolic profile in these commonly used control strains with those observed in the fed C57BL/6 mice.

### MRS data analysis

All cardiac ^13^C spectra were analysed using the AMARES algorithm in the jMRUI software package [36]. Spectra were DC offset-corrected based on the last half of acquired points. The peak areas of [1-^13^C]pyruvate, [1-^13^C]lactate, [1-^13^C]alanine and ^13^C]bicarbonate at each time point were quantified and used as input data for a kinetic model. The kinetic model developed for the analysis of hyperpolarized [1-^13^C]pyruvate data is based on a model developed by [19,37]*.*

### Statistics

All results are expressed as mean ± SEM. Significant differences between mean values were determined by one-way analysis of variance (ANOVA) followed by Bonferroni’s multiple comparison post-hoc test. Differences between groups were considered significant if p < 0.05.

## Results

### Acquisition of [1-^13^C]pyruvate in the in vivo mouse heart

**Figure 2:**
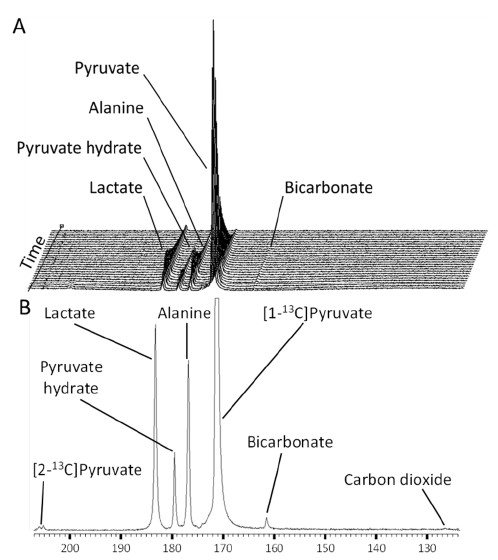
**Example mouse spectra. A**) Example spectra in a fed wild type C57BL/6 mouse, every other second is represented for clarity. **B**) Summed spectrum of 40 individual spectra from a fed wild type C57BL/6 mouse. N.B. [2-^13^C]pyruvate is derived from ^13^C natural abundance pyruvate.

A typical set of spectra from a fed control mouse is shown in Figure 2 (C57BL/6). Along with the injected [1-^13^C]pyruvate, the downstream metabolites, [1-^13^C]lactate, [1-^13^C]alanine and [^13^C]bicarbonate can be visualized with 1 second temporal resolution (Figure 2A). [^13^C]bicarbonate is produced from the conversion of ^13^CO_2_ by the carbonic anhydrase enzyme. Due to the low SNR of the peak and a rapid exchange into [^13^C]bicarbonate, ^13^CO_2_ is only visible when the spectra are summed (Figure 2B).

### The appearance of pyruvate and lactate resonance in mouse versus rat

**Figure 3:**
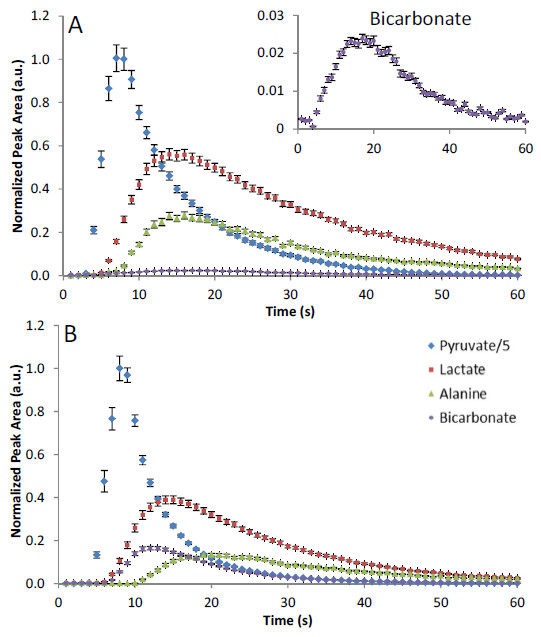
**Example mouse and rat time course.A**) Shows a combined time course in control mouse hearts (C57BL/6), in which the bicarbonate signal is reduced compared to the rat (the insert shows the bicarbonate time course expanded). **B**) Shows a combined time course in the rat (Wistar) for pyruvate and the three main metabolites lactate, alanine and bicarbonate (n=4).

An averaged time course of the fitted peak areas of pyruvate, lactate, alanine and bicarbonate from the fed C57BL/6 mouse heart (n = 5) and the fed rat heart (n = 4) are shown in Figure 3. Curves are normalized to the peak pyruvate signal intensity to account for any differences in the initial polarization and coil sensitivity. Alanine appeared significantly earlier in the mouse heart, compared to the rat heart (2.6 ± 0.8 s vs 6 ± 1 s, time of arrival normalized to pyruvate arrival, p < 0.05), bicarbonate also appeared significantly earlier in the mouse heart (1.4 ± 0.2 s vs 2.3 ± 0.3 s, time of arrival normalized to pyruvate arrival, p < 0.05). Maximum bicarbonate signal was reduced 7-fold in the mouse heart compared to the rat heart (0.023 ± 0.001 a.u. vs 0.165 ± 0.008 a.u. bicarbonate normalized to peak pyruvate signal). This may represent a difference in PDH activity in the mouse heart compared to the rat heart.

### Overnight fasting and DCA modulate PDH flux in vivo

**Figure 4:**
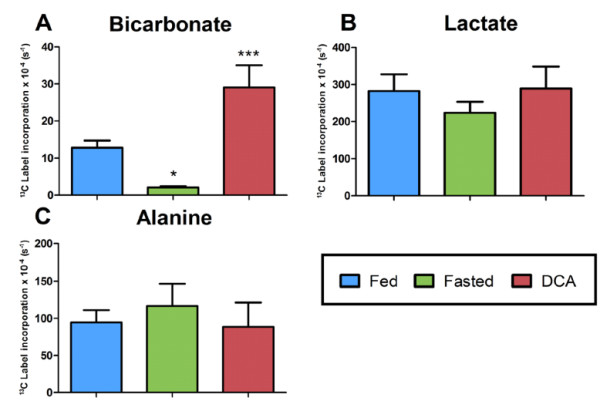
**Demonstration of hyperpolarized [1-**^**13**^**C]pyruvate in the in vivo mouse heart. A**) Fasting in control mice significantly decreased ^13^C label incorporation into bicarbonate in the C57BL/6 mouse heart, compared to fed controls. Infusion of DCA significantly increase in ^13^C label incorporation compared to fed mice. **B**) No difference was found in ^13^C label incorporation of lactate between fed, fasting and DCA infusion. **C**) No significant difference was found between ^13^C label incorporation into alanine in the fed, fasted or DCA state. All results are expressed as the mean **±** SEM. * p < 0.05 and *** p < 0.001 compared to fed controls.

To define the sensitivity of the DNP technique in mice, two well characterized models of PDH flux modulation were employed (Figure 4). Overnight fasting led to a significant 85% reduction in ^13^C label incorporation into bicarbonate compared to fed controls (from 13 ± 2 (× 10^-4^) s^-1^ to 2.0 ± 0.5 (× 10^-4^) s^-1^, p < 0.05). No significant change was observed in the ^13^C label incorporation into lactate (from 300 ± 50 (× 10^-4^) s^-1^ to 225 ± 25 (× 10^-4^) s^-1^) or ^13^C label incorporation into alanine (from 90 ± 20 (× 10^-4^) s^-1^ to 130 ± 25 (× 10^-4^) s^-1^). Infusion of 0.3 ml of 30 mg/kg DCA significantly increased PDH flux by 123% (from 13 ± 2 (× 10^-4^) s^-1^ to 29 ± 6 (× 10^-4^) s^-1^, p < 0.001). Again no differences in ^13^C label incorporation into lactate or alanine were observed.

### Comparison of commonly used mouse strains

**Figure 5:**
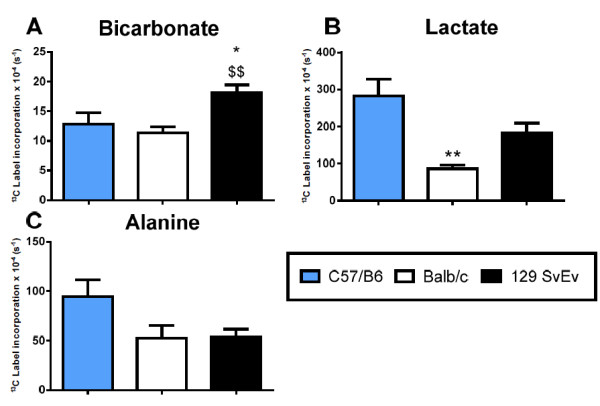
**Comparison between different mouse strains.** Several commonly used mouse strains (C57BL/6, Balb/c and 129 SvEv) were compared using [1-^13^C]pyruvate. **A**) Label incorporation into bicarbonate was found to be similar between the C57BL/6 and balb/c, it was however significantly elevated in the 129 SvEv. **B**) Label incorporation into lactate was significantly reduced in the balb/c mouse, compared to C57BL/6. **C**) No difference was seen in alanine. All results are expressed as the mean **±** SEM. * p < 0.05 and ** p < 0.01 vs C57BL/6 and $$ p < 0.01 compared to Balb/c.

Following the successful demonstration of the sensitivity of the mouse DNP technique, comparison of the metabolic profiles of the commonly used control strains, balb/c and 129 SvEv were compared to the data from fed C57BL/6 mice (Figure 5). ^13^C label incorporation into bicarbonate was significantly higher in the 129 SvEv, compared to both balb/c and C57BL/6 mice. Label incorporation into lactate was significantly lower in the balb/c mice compared to C57BL/6. ^13^C label incorporation into alanine appears elevated in the C57BL/6 mice, although this failed to reach statistical significance, (C57BL/6 verses 129 SvEv p=0.06).

## Discussion

In this study, we have developed a hyperpolarized MRS technique for the assessment of cardiac metabolism in the in vivo mouse heart, using a custom built ^13^C surface transmit/receive coil. The development of mouse cardiac DNP provided several challenges, including the requirement of a small ^13^C surface transmit/receive coil, which due to the reduction in size of the mouse heart, when compared to the rat heart, was required to enable localization of signal to the heart and provide sufficient SNR. It was also necessary to ensure that acquisitions were ECG gated to account for the increased motion and speed of the mouse heart. The final challenge was creating an injection line, which would allow the delivery of the hyperpolarized fluid, whilst not overloading the mouse’s venous system, thereby allowing serial timepoint measurements. Using ultra fine bore tubing and an insulin needle, the dead volume in the cannula was reduced to approximately 50 μl.

As expected, spectra acquired in wild type mice (C57BL/6) showed similar resonances to the rat. However, carbon dioxide was only occasionally visible in the high temporal resolution spectra. Carbon dioxide was present in summed spectra, indicating that its absence was due to lower SNR in the mouse compared to the rat. Pyruvate and lactate appearance mirrored that of rats, whilst alanine signals appeared to be slightly elevated compared to the time evolution in the rat and also showed significant temporal differences in appearance. However, the major difference between rat and mouse spectra was the relative levels of bicarbonate production, which were 7-fold lower in the mouse heart compared to the rat heart when normalized to maximum pyruvate signal, possibly representing a species difference in PDH expression/activity due to differences in cardiac work load. This is supported by previous measurements which would suggest a lower PDH activity in the mouse compared with the rat (2.4 μmol/min/g tissue (mouse) [38] vs 5.1 μmol/min/g tissue (rat) [19]).

In this work, the sensitivity of the cardiac hyperpolarized MRS technique was demonstrated to detect in vivo alterations in PDH flux. Using hyperpolarized [1-^13^C]pyruvate in the rat, a significant reduction in cardiac PDH flux was previously observed in the fasted state [14]. Further, infusing 30 mg/kg of DCA was shown to significantly increase PDH flux in Wistar rat hearts [19]. Both methods of PDH modulation rely on alterations to the inhibitor of PDH, PDH kinase (PDK) [39,40] (reviewed in [41]). During fasting, plasma fatty acid levels increase, insulin levels drop and fatty acid oxidation increases in the heart [14,42,43]. In response, PPARα is activated and increases the expression of PDK4, which acts to “spare” pyruvate for oxaloacetate formation [42,44-46]. In rats, this led to a significant 74% reduction in PDH flux, seen as a drop in the total ^13^C]bicarbonate/[1-^13^C]pyruvate ratio [14]. In this study, mice fasted overnight for a minimum of 19 hours had an 85% decrease in PDH flux compared to control the fed state group (p < 0.05).

The third group of mice received a DCA infusion prior to the hyperpolarized injection. DCA has a broad inhibitory action on all 4 isoforms of PDK, thereby increasing the proportion of PDH in the active form [39,40,47]. In rats, DCA infusion resulted in a 160% increase in PDH flux in control animals [19]. In mice the infusion of DCA significantly increased PDH flux by 123%, compared to control mice (p < 0.001). Evaluation of the mouse DNP technique proved it was sensitive to alterations in PDH flux within the mouse heart and demonstrated the potential to use hyperpolarized MRS to investigate transgenic mouse models of cardiac diseases in the future.

Following demonstration of the technique, we characterized the metabolic profile of other commonly used control mouse strains. C57BL/6, 129 SvEv and balb/c mice are inbred mice strains used for metabolic studies and are commonly used as breeding backgrounds for transgenic mouse lines [29,30]. In mouse models of heart disease, observed differences in metabolic profiles are influenced by the background strain used to generate the mouse [48,49]. In the study by Gavaghan et al. [49], NMR analysis of control strain urine found that there were differences in metabolite composition between healthy mouse strains. In our study several differences were observed in cardiac ^13^C label incorporation into metabolite pools, between these control strains. The 129 SvEv strain had elevated ^13^C label incorporation into bicarbonate, suggesting a difference in PDH flux, when compared to C57BL/6 and balb/c mice. ^13^C label incorporation into lactate was reduced in the balb/c strain compared to C57BL/6, but not compared to 129 SvEv. The strain differences presented in this paper are supported by significant differences in glycolytic and glucose oxidation rates and glucose uptake presented in previous work from these three strains [29,50-52]. These differences highlight the important requirement of selecting both the correct mouse background and appropriate control animals for metabolic studies.

## Conclusion

In conclusion, we have successfully developed and demonstrated a hyperpolarized DNP protocol to investigate alterations in metabolism within the in vivo mouse heart. The use of fasting and DCA has demonstrated the sensitivity of the technique to detect alterations in PDH activity in the mouse heart. The technique has also highlighted possible metabolic differences in strains of mice commonly used for metabolic studies. This technique offers significant advantages over existing in vitro techniques that would require collection of tissue samples, as it can reduce animal numbers and decrease biological variability. Thus [1-^13^C]pyruvate can be used to monitor the in vivo cardiac metabolic profile of transgenic mice.

## Competing interests

The authors declare that they have no competing interests.

## Authors’ contributions

MSD carried out the data acquisition, data analysis and manuscript preparation. VB and RB carried out data acquisition and manuscript editing. HA, HW and KC provided experimental design and editorial assistance with the manuscript. DJT conceived and coordinated the study, and edited the manuscript. All authors read and approved the final manuscript.

## Disclosures

Equipment support was provided by General Electric Healthcare (Amersham, UK).
